# Causal effects of antibody-mediated immunity to pathogens on five ophthalmic diseases: a Mendelian randomization study

**DOI:** 10.3389/ebm.2026.10906

**Published:** 2026-01-30

**Authors:** Liduo Pan, Jian Deng, Jianli Yang, Meng Wang, Zheng Chen, Tao Wang, Yonghao Li

**Affiliations:** 1 Department of Ophthalmology, Aier Eye Hospital, Jinan University, Guangzhou, Guangdong, China; 2 Department of Ophthalmology, The Third Affiliated Hospital of Sun Yat-sen University, Guangzhou, Guangdong, China; 3 Department of Ophthalmology, Shenzhen Eye Hospital, Shenzhen Eye Medical Center, Southern Medical University, Shenzhen, Guangdong, China

**Keywords:** Mendelian randomization, infection-induced immune response, chronic iridocyclitis, scleritis, wet age-related macular degeneration, diabetic retinopathy, glaucoma

## Abstract

While immune responses related to infections have been linked to ocular diseases, their causal role remains to be established. This study aimed to assess the causal relationship between antibody-mediated immune responses to infectious agents and five ocular conditions: chronic iridocyclitis (CIR), scleritis, wet age-related macular degeneration (AMD), diabetic retinopathy (DR), and glaucoma. We performed a two-sample Mendelian randomization (MR) analysis using GWAS data to assess causality between antibody responses to 46 pathogens and five ophthalmic diseases. The instrumental variables were Single nucleotide polymorphisms (SNPs). Causal estimates were primarily generated via the inverse-variance weighted method, supplemented by MR-Egger and weighted median methods. A Bonferroni-corrected threshold of P < 2.17 × 10^−4^ was applied. Sensitivity analyses included Cochran’s Q, MR-Egger, and MR-PRESSO for heterogeneity and pleiotropy. Reverse MR was performed to assess bidirectionality. Forward MR identified causal effects of infection-induced immune responses on ocular diseases. Epstein-Barr virus (EBV) ZEBRA antibodies were positively correlated with CIR, whereas Varicella zoster virus glycoproteins E and I antibodies were associated with scleritis and DR as risk factors. Genetically predicted anti-polyomavirus 2 IgG seropositivity (JCV IgG+) was identified as a risk factor for DR, wet AMD and glaucoma. In contrast, The EBV EBNA-1 antibody is associated with DR, wet AMD, and glaucoma as a protective factor, whereas the EBV VCA18 antibody is negatively associated with wet AMD. Reverse MR analysis indicated that DR may elevate JCV VP1 antibody levels. This study provides the first genetic evidence of a causal link between pathogen-specific immune responses and ocular diseases, offering a foundation for targeted immunomodulatory and personalized therapies.

## Impact statement

This research systematically elucidates, via Mendelian randomization, the causal relationships between pathogen-specific antibody responses and five significant ophthalmic diseases, offering essential genetic evidence for the discipline. This study confirms the significant role of infection-induced immune responses in the pathogenesis of chronic eye diseases and elucidates the specific mechanisms of viruses such as EBV, VZV, and JCV in various ocular conditions. It shifts the paradigm from traditional risk factors to infection-related immune mechanisms in understanding disease etiology. The findings provide a theoretical basis for immunological prevention and personalized treatment of ophthalmic diseases, particularly facilitating the development of targeted interventions against specific pathogen-induced immune responses.

## Introduction

Chronic ocular conditions, including chronic iridocyclitis (CIR), scleritis, age-related macular degeneration (AMD), diabetic retinopathy (DR), and glaucoma, are significant contributors to vision impairment and blindness globally [1–5]. These diseases considerably reduce patients’ quality of life and impose a significant societal burden. Despite their distinct clinical manifestations and pathological features, increasing evidence suggests that immune dysregulation and chronic inflammation constitute a common pathophysiological basis among these conditions [6, 7].

Immune responses are fundamental to the pathogenesis of the ocular diseases discussed. Iridocyclitis and scleritis are frequently associated with systemic autoimmune disorders, including rheumatoid arthritis and systemic lupus erythematosus [8, 9]. The pathogenesis encompasses infection, immune dysregulation, inflammatory processes, and genetic susceptibility, with immune reactivity serving as a pivotal driving factor [10]. Significant immune and inflammatory mechanisms have been identified in diseases traditionally attributed to vascular or metabolic abnormalities. Dysregulated immunity, microangiopathy, and metabolic disturbances collectively contribute to disease development in DR [11]. AMD is a neurodegenerative condition that impacts the eye. Alongside mitochondrial dysfunction, oxidative stress, and autophagy defects, immune-mediated inflammatory conditions are recognized as factors associated with AMD [12, 13]. Additionally, in glaucoma, a neurodegenerative condition marked by irreversible vision loss resulting from optic nerve atrophy and retinal ganglion cells (RGCs) death, dysregulated immune signaling and T-cell-mediated autoimmunity are implicate [14].

Infectious factors are gaining attention as a potential etiological mechanism for the previously mentioned ocular diseases [15, 16]. Pathogens can induce systemic immune and inflammatory responses; thus, chronic or latent infections may lead to ocular pathology through immune-mediated mechanisms. For example, Epstein-Barr virus specific immune responses have been linked to autoimmune uveitis [17], and ocular herpes zoster virus infection can lead to a range of conditions such as conjunctivitis, uveitis, episcleritis, keratitis, and retinitis [18]. Additionally, research indicates that viral infections may aggravate ocular neurodegeneration by triggering neuroinflammation and disturbing neuronal protein homeostasis [19].

Nevertheless, despite an exhaustive search, no studies have definitively established a causal link between antibody-mediated immune responses—particularly those triggered by infection—and ocular diseases. There is therefore a clear need for robust causal inference approaches to clarify these associations. Mendelian randomization (MR), an epidemiological technique that utilizes genetic variants as instrumental variables, can elucidate these relationships while minimizing confounding and reverse causality [20, 21].

This study aims to utilize two-sample Mendelian randomization analysis to examine the causality between 46 antibody-mediated immune traits and five ocular disorders: chronic iridocyclitis, scleritis, wet age-related macular degeneration, diabetic retinopathy, and glaucoma.

## Materials and methods

### Data selection and sources

To assess causality, we conducted a Mendelian randomization analysis using genetically predicted antibody responses to infections as exposures and the five ocular diseases as outcomes, selecting associated SNPs as instrumental variables. Summary data on antibody-mediated immune responses were obtained from the GWAS Catalog.^1^ These data originated from the study by Butler-Laporte et al. [22], who performed serological profiling on 9,724 adults of European ancestry from the UK Biobank using 13 pathogens to define 46 phenotypes. Details of all phenotypes are provided in Supplementary Table S1. Summary statistics for the five ocular diseases were sourced from the FinnGen GWAS.^2^ All participants were of European ancestry. Case and control numbers were as follows: chronic iridocyclitis (1,869/473,095), scleritis (441/473,095), neovascular AMD (6,699/331,070), diabetic retinopathy (14,142/82,287), and glaucoma (26,591/473,757). Details of the case and control groups for each eye disease are shown in Supplementary Table S2.

### Instrumental variable selection

**FIGURE 1:**
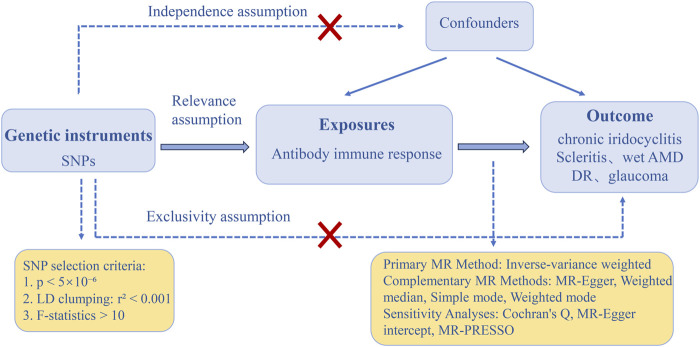
The three main assumptions of Mendelian randomization. DR, diabetic retinopathy; Wet AMD, Wet age-related macular degeneration; SNPs: single-nucleotide polymorphisms; MR, Mendelian randomization; LD, linkage disequilibrium; MR-PRESSO, Mendelian randomization-pleiotropy residual sum and outlier.

In our MR analysis, single nucleotide polymorphisms (SNPs) served as instrumental variables (IVs), following three critical assumptions: (1) association with the exposure, (2) independence from confounders, and (3) influence on the outcome exclusively through the exposure (Figure 1). Rigorous selection of instrumental variables (IVs) is essential for ensuring the robustness of Mendelian randomization (MR) analysis. Initially, instrumental variables (IVs) must exhibit a robust association with the exposure, with the standard threshold for genome-wide significance established at p < 5 × 10^−8^. To maintain an adequate number of instrumental variables for each phenotype, we adjusted the threshold to p < 5 × 10^−6^. A screening procedure was conducted to eliminate the effects of linkage disequilibrium (LD), confirming that the chosen SNPs displayed low LD (r^2^ < 0.001 within a 10,000 kb window) [23].

The strength of the IVs was assessed using the F-statistic, defined as $F=R2\;\left(N-K-1\right)/K\left(1-R2\right)$ . R^2^ is defined as the proportion of variance in exposure explained, where N denotes the sample size and K indicates the number of instruments. The formula for calculating R^2^ is as follows: R^2=2×MAF×1−MAF×β/SD^2 . In the equation, MAF represents the minor allele frequency, β denotes the effect size, and SD indicates the standard deviation [24]. To rule out weak instrument bias, all included SNPs were ensured to have F-statistics greater than 10.

### MR analysis

We conducted forward MR analyses to evaluate causal relationships between genetically predicted infection-related antibody responses and five ocular diseases, employing five methodologies: inverse-variance weighted (IVW), MR-Egger, weighted median, simple mode, and weighted mode. The IVW method was selected as the primary approach due to its assumption of valid instruments without horizontal pleiotropy, while the other methods functioned as complementary analyses [25]. All analyses were performed using R (version 4.5.1) with the TwoSampleMR package (version 0.6.22). The presence of 46 immune phenotypes associated with five ocular diseases as outcomes heightens the likelihood of Type I errors due to repeated testing. Consequently, we utilized Bonferroni correction, which is a more rigorous method than FDR adjustment, for multiple significance testing. A p-value of less than 2.17 × 10^−4^ (0.05/46/5) was considered statistically significant.

Reverse MR was conducted to assess the causal relationships between five ocular diseases (as exposures) and antibody-mediated immune responses (as outcomes), adhering to an analytical procedure aligned with the forward MR approach. The same Bonferroni correction method is employed.

### Sensitivity analysis

To evaluate the validity of instrumental variables and robustness of the results, we conducted comprehensive sensitivity analyses. Heterogeneity was assessed using Cochran’s Q statistic from IVW and MR-Egger regression, with P > 0.05 indicating no significant heterogeneity [26]. Horizontal pleiotropy was examined via the MR-Egger intercept test, where P > 0.05 suggested no evidence of directional pleiotropy, and the MR-PRESSO global test was applied to identify and remove outliers. If outliers existed, the MR analysis was repeated after eliminating these outliers [27, 28]. Additionally, leave-one-out analyses were performed to assess whether overall associations were driven by individual influential variants and to visualize the contribution of each SNP. To evaluate the overall robustness, funnel plots were generated using R (version 4.5.1) to assess the symmetry of the SNPs.

## Results

The MR analysis indicates that various virus-mediated antigen-antibody responses have a genetically predicted causal association with the risk of developing ocular diseases.

### Risk effects of infection-related antibody responses

Based on forward MR analyses, Epstein-Barr virus (EBV) ZEBRA antibody levels, varicella-zoster virus glycoprotein E and I (VZV gE & gI Ab) antibody levels, and anti–JC virus IgG seropositivity (JCV IgG+) were identified as risk factors for multiple ocular diseases. The results of sensitivity analyses and visualisations are presented in Supplementary Table S3, Supplementary Figures S1, S2.

#### Causal effect of EBV ZEBRA antibodies on chronic iridocyclitis

**FIGURE 2:**
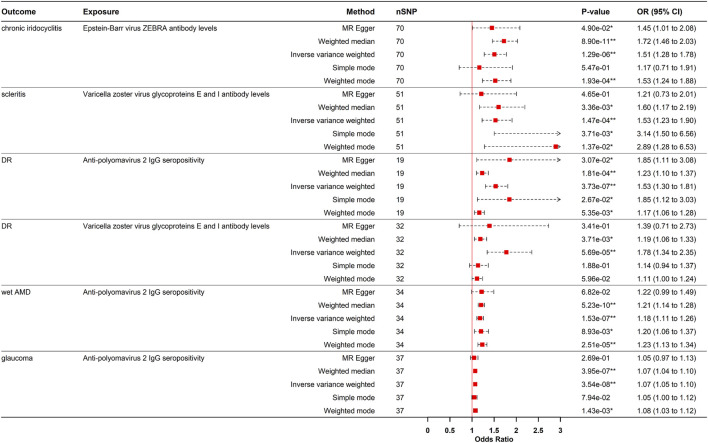
Forest plot visualization of the Positive causal effect of antibody-mediated immune responses on five eye diseases. DR, diabetic retinopathy; Wet AMD, Wet age-related macular degeneration; MR, Mendelian randomization; SNPs, single-nucleotide polymorphisms; OR, odds ratio; CI, confidence interval; *p- value <0.05; **p- value <0.00021.

**FIGURE 3:**
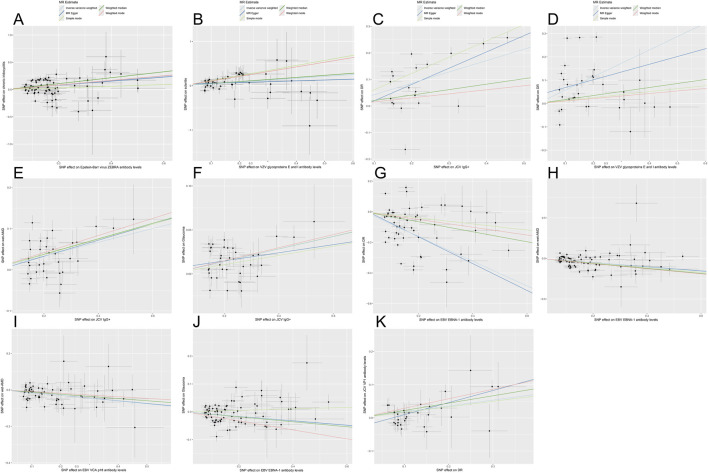
Scatter plots for the causal relationship of antibody-mediated immune responses and five eye diseases. Scatterplots demonstrate the influence of SNPs on different parameters using five MR methods: inverse variance weighting, MR-Egger, simple mode, weighted median, and weighted mode. Each subplot reflects the relationship between SNP effects on the horizontal axis (immunophenotype) and ophthalmic diseases on the vertical axis. Black dots represent specific SNP effect estimates, whereas gray bands indicate the uncertainty linked to these estimates. The results of linear regression for the 5 methods are shown by lines of different colors. Panels correspond to: **(A)** Epstein-Barr virus (EBV) ZEBRA antibody with chronic iridocyclitis (CIR); **(B)** Varicella zoster virus (VZV) glycoprotein E & I antibody with scleritis; **(C)** Anti-polyomavirus 2 IgG seropositivity (JCV IgG+) with diabetic retinopathy (DR); **(D)** VZV gE & gI antibody with DR; **(E)** JCV IgG+ with wet age-related macular degeneration (AMD); **(F)** JCV IgG+ with glaucoma; **(G)** EBV EBNA-1 antibody with DR; **(H)** EBV EBNA-1 antibody with wet AMD; **(I)** EBV VCA p18 antibody with wet AMD; **(J)** EBV EBNA-1 antibody with glaucoma; **(K)** Reverse MR analysis for DR with JCV VP1 antibody.

Genetically predicted levels of EBV ZEBRA antibodies levels increased the risk of CIR (OR = 1.506, 95% CI: 1.276–1.778, PIVW = 1.292e-06). This finding was consistent using weighted median and weighted mode methods (Figures 2, 3). Although sensitivity analyses indicated heterogeneity, no horizontal pleiotropy was detected by the MR-Egger intercept test. The association remained significant after outlier removal via MR-PRESSO and was robust in leave-one-out analysis, collectively supporting the reliability of the causal inference.

#### Causal effects of VZV gE & gI antibodies on scleritis and DR

VZV gE & gI antibody levels were causal risk factors for both scleritis (OR = 1.529, 95% CI: 1.228–1.904, p = 1.473e-04) and DR (OR = 1.776, 95% CI: 1.343–2.350, P_IVW_ = 5.689e-05) (Figures 2, 3). For scleritis, no heterogeneity or horizontal pleiotropy was detected. Cochran’s Q test showed that there was heterogeneity for DR, but no horizontal pleiotropy was found. Both causal estimates remained significant after MR-PRESSO outlier removal and were robust in leave-one-out analyses.

#### Causal effects of JCV IgG+ on DR, wet AMD, and glaucoma

Additionally, genetically predicted JCV IgG+ significantly increased the risks of DR (OR = 1.533, 95% CI: 1.300–1.808, P_IVW_ = 3.733e-07), wet AMD (OR = 1.182, 95% CI: 1.110–1.258, P_IVW_ = 1.529e-07), and glaucoma (OR = 1.073, 95% CI: 1.046–1.100, P_IVW_ = 3.537e-08) (Figures 2, 3). These causal estimates were consistently supported by the weighted median method and showed no evidence of horizontal pleiotropy. Despite some heterogeneity, the causality remained significant after outlier removal and were robust in leave-one-out sensitivity analyses, confirming the stability of the results.

### Protective effects of infection-related antibody responses

#### Causal effects of EBV EBNA-1 antibodies on DR, wet AMD, and glaucoma

Conversely, genetically predicted EBV EBNA-1 antibody levels causally reduced the risk of DR (OR = 0.445, 95% CI: 0.389–0.532, P_IVW_ = 1.019e-22), wet AMD (OR = 0.778, 95% CI: 0.720–0.840, P_IVW_ = 1.343e-10), and glaucoma (OR = 0.922, 95% CI: 0.889–0.956, P_IVW_ = 1.675e-05).

#### Causal effect of EBV VCA p18 antibodies on wet AMD

**FIGURE 4:**
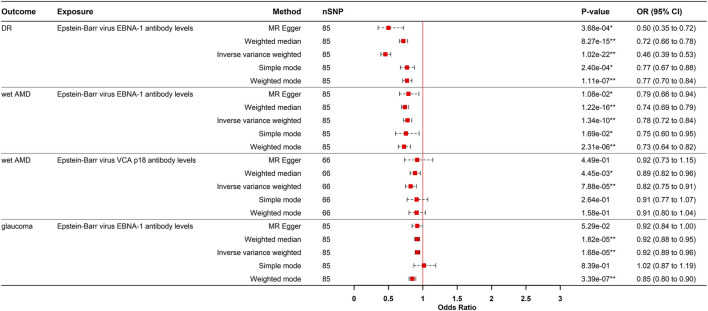
Forest plot visualization of the negative causal effect of antibody-mediated immune responses on five eye diseases. DR, diabetic retinopathy; Wet AMD, Wet age-related macular degeneration; MR, Mendelian randomization; SNPs, single-nucleotide polymorphisms; OR, odds ratio, CI, confidence interval; *p- value <0.05; **p- value <0.00021.

Similarly, genetically predicted EBV VCA p18 antibody levels were a protective factor against wet AMD (OR = 0.822, 95% CI: 0.746–0.906, P_IVW_ = 7.875e-05). These causal relationship were consistently supported by weighted median and weighted mode methods (Figures 3, 4). While significant heterogeneity was observed, MR-Egger intercept tests revealed no directional pleiotropy. The results remained significant following MR-PRESSO outlier correction, confirming the stability of these protective relationships (Supplementary Table S3). The leave-one-out sensitivity analysis and funnel plot confirmed the robustness of the results (Supplementary Figures S1, S2).

### Reverse MR analysis

**FIGURE 5:**
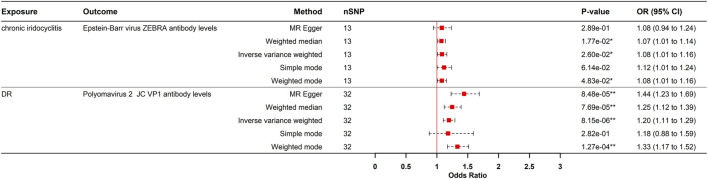
Causal effects of eye diseases on antibody-mediated immune responses in the reverse MR analysis. DR, diabetic retinopathy; MR, Mendelian randomization; SNPs, single-nucleotide polymorphisms; OR, odds ratio; CI, confidence interval; *p- value <0.05; **p- value <0.00021.

To evaluate reverse causation, we performed reverse MR analyses to assess the effects of ocular diseases on viral antibody levels. DR had a positive causal effect on JCV VP1 antibody levels after strict multiple testing correction (OR = 1.195, 95% CI: 1.105–1.292, P = 8.150e-06), corresponding to a 19.5% increased risk of elevated JCV VP1 antibodies in DR patients. Consistency across MR-Egger, weighted median, IVW, and weighted mode methods strengthened causal credibility (Figures 3, 5). While the MR-Egger intercept indicated potential minor horizontal pleiotropy, neither the MR-PRESSO nor Cochran’s Q test showed significance (Supplementary Table S3; Supplementary Figures S1, S2).

A nominally significant causal relationship was observed between CIR and EBV ZEBRA antibody levels (OR = 1.082, 95% CI: 1.009–1.161, *P* = 0.025). However, this association did not survive strict multiple testing correction (Bonferroni-corrected threshold *P* < 0.0021) and should therefore be interpreted with caution.

## Discussion

This bidirectional MR study provides genetic evidence supporting causal relationships between infection-related antibody responses and ocular disorders. Specifically, elevated EBV ZEBRA, VZV gE & gI, and JCV IgG+ antibody levels increased the risk of chronic iridocyclitis (CIR), scleritis, DR, wet AMD, and glaucoma, whereas EBV EBNA-1 and VCA p18 antibodies exhibited protective effects. Reverse MR analysis further indicated that DR contributes to higher JCV VP1 antibody levels.

### Potential mechanisms connecting EBV ZEBRA antibodies to CIR

EBV is a prevalent pathogen, infecting more than 90% of the global population, and is linked to several autoimmune diseases and malignancies [29]. We present genetic evidence indicating that the ZEBRA antibody, derived from the immediate-early gene BZLF1 that triggers the viral lytic cascade, causally increases the risk of CIR through various potential mechanisms.

One key mechanism is molecular mimicry. EBV proteins contain epitopes that resemble human proteins, and viral reactivation causes cell death and self-antigen release [30]. Upon presentation by antigen-presenting cells (APCs), these antigens may activate autoreactive T cells, which can cross-react with ocular tissues and trigger iridocyclitis. Studies have shown that in uveitis patients, Th1 cells activate macrophages via IFN-γ, while Th17 cells sustain chronic inflammation through IL-17. Together, these responses promote leukocyte recruitment and local inflammatory cascades, ultimately leading to tissue damage via oxidative stress and pro-inflammatory cytokine release [31, 32].

Secondly, by modulating signaling pathways, EBV promotes B cell differentiation into T-bet^+^ B cells [30]. These cells subsequently produce pathogenic IgG1/IgG3 autoantibodies and IFN-γ, establishing a pro-inflammatory feedback loop [33]. Their additional role in disrupting tissue barriers may permit CXCR3^+^ B cell migration across the blood-aqueous barrier, initiating ocular inflammation. This is corroborated by the marked increase in IL-6 and chemokines (CXCL13, CCL8, CCL13, CCL20) in the aqueous humor of uveitis patients [34].

Additionally, the EBV early lytic protein BALF0/1 hijacks host caveolin-mediated endocytosis and ERAD pathways during viral replication to degrade the B-cell receptor complex [35]. Thus, BZLF1 (ZEBRA)-driven promoter activation likely upregulates BALF0/1, disrupting B-cell function, promoting viral release, and ultimately causing local ocular immune dysregulation that contributes to chronic iridocyclitis.

The link between EBV specific immune responses and autoimmune uveitis is further supported by Hendrikse et al. [17], who observed elevated antibody levels against the RRPFFHPV motif of Epstein–Barr virus nuclear antigen 1 (EBNA-1) in pediatric uveitis patients carrying the *HLA-DRB1*15:01* risk allele, with the strongest IgG signals detected in those with non-anterior uveitis. This strongly suggests a role for EBNA-1–directed immunity in certain forms of autoimmune uveitis. In contrast, our analysis supports EBV EBNA-1 antibody levels as a protective factor against DR, wet AMD, and glaucoma. We thus propose that beyond its potential role as an initial trigger or cross-reactive target, EBNA-1 may also induce a state of immune tolerance or modulate dominant inflammatory pathways, thereby conferring a protective effect in these ocular disorders.

### The protective function of EBV EBNA-1 antibodies in DR, wet AMD, and glaucoma

EBV may exhibit differential impacts on ocular diseases depending on the stage of the viral life cycle. In our study, latent infection marked by EBNA-1 antibodies conferred protection against DR, wet AMD, and glaucoma, implicating a modulatory role in the chronic immune dysregulation common to these diseases. Chronic inflammation and immune dysregulation are fundamental pathogenic drivers common to these three conditions [36, 37]. In DR and wet AMD, activated glial cells and macrophages secrete critical mediators including VEGF, TNF-α, IL-1β, and IL-6, which facilitate inflammation and angiogenesis [11, 38]. In glaucoma, T-cell infiltration and microglial activation play a role in the loss of RGCs, which is further aggravated by cytokines such as TNF-α, IL-6, and IL-8 [39, 40]. The protective role of EBNA-1 indicates its potential involvement in the modulation of inflammatory pathways.

Research indicates that EBNA-1 can suppress the canonical NF-κB pathway by inhibiting phosphorylation of IKKα/β [41]—a key signaling axis driving inflammation in diseases such as DR and AMD. By dampening NF-κB activation, EBNA-1 may reduce the expression of pro-inflammatory genes, albeit in a manner that also favors viral persistence and oncogenesis. Furthermore, latent EBNA-1 infection evades cytotoxic immune responses and may promote immune tolerance, possibly through the induction of regulatory T cells or specific antibody subtypes that mitigate auto-reactive immunity against ocular antigens [42]. This modulated immune environment may also restrain excessive macrophage and microglial activation in the retina and choroid [43], thereby limiting VEGF and pro-inflammatory cytokine production and potentially slowing disease progression.

### Pathogenic mechanisms of JCV IgG+ in DR, wet AMD, and glaucoma

Conversely, our analysis supports a causal role for JCV IgG+ seropositivity in DR, age-AMD, and glaucoma. This ubiquitous virus, which persists asymptomatically in most adults, may reactivate under immunosuppression, enter circulation, and promote ocular neurovascular pathology [44, 45]. Critically, studies have confirmed that JCV can infect human cerebral vascular pericytes to breach the blood-brain barrier and subsequently invade astrocytes, causing neural damage [46]. As an extension of the central nervous system, the retina shares key developmental and structural features with the brain, and its blood-retinal barrier (BRB) relies on pericytes and Müller cells as essential components. Therefore, JCV may similarly target the BRB through comparable mechanisms, contributing to the vascular leakage pathology observed in DR and AMD.

Beyond direct viral invasion, virus-induced immune responses may constitute a key pathogenic mechanism. Murinello et al. [47] observed co-deposition of IgG, C1q, and membrane attack complexes in early AMD eyes, along with increased numbers of FcγRIIa- and FcγRIIb-expressing inflammatory cells in the choroid. Thus, JCV-specific antibodies may form local immune complexes in the retina, activating macrophages or microglia via Fcγ receptors and thereby driving chronic inflammation. Neurotropic viruses such as VZV and HSV-1 promote Aβ aggregation—a key component of AMD-associated drusen—through oxidative stress, calcium dysregulation, and impaired autophagy [48, 49]. As a neurotropic virus, JCV may similarly enhance AMD susceptibility via analogous Aβ-inducing pathways. In glaucoma, JCV could directly damage RGCs, supported by detections of JCPyV DNA and viral inclusions in intraocular fluid and RGCs [50]. These proposed mechanisms, however, await confirmation through larger clinicopathological studies.

### The role of VZV gE and gI antibodies in the pathogenesis of scleritis and DR

Moreover, genetically predicted levels of VZV gE & gI antibodies showed positive causal associations with scleritis and DR. VZV, a double-stranded DNA virus, establishes latency in trigeminal ganglia after primary infection. Upon immunosuppression, reactivated virus can travel along axons to the eye, causing herpes zoster ophthalmicus, which clinically manifests as uveitis, scleritis, or retinal and optic nerve inflammation [18, 51].

VZV may increase susceptibility to DR and scleritis by targeting ocular vasculature. Studies demonstrate that VZV infects vessels via the ophthalmic branch of the trigeminal nerve, subsequently spreading trans-adventitially, disrupting the internal elastic lamina, and triggering intimal hyperplasia and a pro-inflammatory state [52].

Furthermore, the VZV glycoprotein gE acts as a key regulator of PINK1/Parkin-dependent mitophagy. By interacting with the autophagy protein LC3, gE induces substantial mitochondrial reactive oxygen species (mtROS) production [53]. Given that oxidative stress and mitochondrial dysfunction are central to DR pathogenesis, VZV likely exacerbates DR progression by modulating the PINK1/Parkin pathway.

Immune dysregulation further contributes to disease pathogenesis. VZV-induced mitophagy suppresses interferon production by inhibiting the STING and MAVS pathways, leading to elevated pro-inflammatory cytokines and NLRP3 inflammasome activation [53]. The release of these mediators attacks the vascular sclera and compromises retinal vascular stability. Clinical evidence confirms VZV as a cause of scleritis, as demonstrated by the detection of viral DNA in aqueous humour. [16, 54]. The efficacy of immunosuppressants such as methotrexate in some scleritis patients further supports the role of VZV-driven immune dysregulation in disease progression.

### Bidirectional causal relationship between DR and JCV infection

Reverse MR analysis indicates a genetically determined positive causal relationship between DR and JCV VP1 antibody levels. Critically, This finding indicates that genetic susceptibility to DR may predispose individuals to JCV reactivation by modifying the immune environment, rather than suggesting a direct, acute impact on antibody titers.

In diabetic patients, hyperglycemia/advanced glycation end products impair T-cell and B-cell function, leading to immune dysregulation [11]. The advancement of DR entails the disintegration of the BRB and the liberation of inflammatory mediators, including TNF-α. This state of chronic inflammation and immune dysregulation may create a favorable environment for the reactivation of JCV latent in peripheral organs, such as the kidneys. At the molecular level, studies indicate that NF-κB and C/EBPβ regulate JCV transcription through the κB motif within the viral genome, with both transcription factors being modulated by proinflammatory cytokines [55]. Furthermore, latent viruses in oligodendrocytes and astrocytes can be reactivated by inflammatory stimuli, enabling viral protein expression and subsequent replication [56]. Our findings suggest that JCV infection may worsen ocular pathology via mechanisms like BRB disruption and that the DR microenvironment may facilitate JCV reactivation. This bidirectional relationship, marked by mutual reinforcement, provides a more thorough and dynamic understanding of the intricate interaction between viral infection and ocular disorders.

### Limitations

This study has several limitations. Its generalizability is constrained by the use of genomic data primarily from European ancestry, and importantly, the MR design itself captures the effect of lifelong genetic exposure to antibody levels, which differs from the impact of acute infection or transient serological changes. Moreover, despite thorough sensitivity analyses including Cochran’s Q, MR-Egger, and MR-PRESSO to account for pleiotropy and heterogeneity, residual confounding may still occur. The application of a strict Bonferroni correction may, to a certain degree, elevate the likelihood of Type II errors. Finally, the mechanistic insights regarding specific pathogens are still lacking and require additional experimental and clinical investigation.

## Conclusion

This two-sample MR study establishes genetic evidence for the causal involvement of pathogen-specific immune responses in ocular disorders. We demonstrate that EBV reactivation may contribute to chronic iridocyclitis through molecular mimicry and B-cell dysregulation, while JCV appears to compromise the blood-retinal barrier (BRB) in DR and wet AMD, and increases glaucoma susceptibility via retinal ganglion cell injury. Reactivation of VZV likely exacerbates DR through mitochondrial ROS induction and promotes scleritis by impairing STING/MAVS pathway signaling. In contrast, EBNA-1 exhibits protective effects by suppressing NF-κB-mediated inflammation. Bidirectional MR analysis indicates that DR elevates JC VP1 antibody levels, thereby establishing a mutually reinforcing cycle of disease and viral reactivation.

Collectively, our findings provide the first genetic validation of causal relationships between multiple pathogen-specific immune responses and ocular diseases, revealing underlying mechanisms involving immune dysregulation, mitochondrial dysfunction, and inflammatory activation. These insights offer a foundation for developing targeted immunomodulatory and personalized therapeutic strategies in ophthalmology.

## Data Availability

Publicly available datasets were analyzed in this study. This data can be found here: FinnGen GWAS (https://r12.finngen.fi/).

## Footnotes

1
https://www.ebi.ac.uk/gwas/

2
https://r12.finngen.fi/
