# Supplementary material for: Causal effects of antibody-mediated immunity to pathogens on five ophthalmic diseases: a Mendelian randomization study
Source: Exp Biol Med (Maywood). 2026 Jan 30;251:10906. doi: 10.3389/ebm.2026.10906 (PMC12900781; doi:10.3389/ebm.2026.10906)
Supplement: Supplementary file 1 [file Table1.docx]

**Table S1.** GWAS data sources for selecting instrumental variables corresponding to the 46 immunophenotypes.

| GWASID | Phenotype | SNPs | Sample size | Population | Year | Source |
| --- | --- | --- | --- | --- | --- | --- |
| GCST90006884 | Anti-BK polyomavirus IgG  seropositivity | 9,170,312 | 8735 | European | 2020 | GWAS Catalog |
| GCST90006885 | BK polyomavirus VP1  antibody levels | 9,170,146 | 8555 | European | 2020 | GWAS Catalog |
| GCST90006886 | Anti-chlamydia trachomatis  IgG seropositivity | 9,170,312 | 8735 | European | 2020 | GWAS Catalog |
| GCST90006887 | Chlamydia trachomatis  momp A antibody levels | 9,201,352 | 964 | European | 2020 | GWAS Catalog |
| GCST90006888 | Chlamydia trachomatis  momp D antibody levels | 9,186,659 | 1371 | European | 2020 | GWAS Catalog |
| GCST90006889 | Chlamydia trachomatis  pGP3 antibody levels | 9,177,921 | 1784 | European | 2020 | GWAS Catalog |
| GCST90006890 | Chlamydia trachomatis  PorB antibody levels | 9,138,738 | 273 | European | 2020 | GWAS Catalog |
| GCST90006891 | Chlamydia trachomatis  tarp-D F1 antibody levels | 9,162,340 | 1635 | European | 2020 | GWAS Catalog |
| GCST90006892 | Chlamydia trachomatis  tarp-D F2 antibody levels | 9,167,334 | 2074 | European | 2020 | GWAS Catalog |
| GCST90006893 | Anti-cytomegalovirus IgG  seropositivity | 9,170,312 | 8735 | European | 2020 | GWAS Catalog |
| GCST90006894 | Cytomegalovirus pp28  antibody levels | 9,170,765 | 5087 | European | 2020 | GWAS Catalog |
| GCST90006895 | Cytomegalovirus pp52  antibody levels | 9,172,314 | 5681 | European | 2020 | GWAS Catalog |
| GCST90006896 | Cytomegalovirus pp150  antibody levels | 9,169,705 | 5136 | European | 2020 | GWAS Catalog |
| GCST90006897 | Anti-Epstein-Barr virus IgG  seropositivity | 9,170,312 | 8735 | European | 2020 | GWAS Catalog |
| GCST90006898 | Epstein-Barr virus EA-D  antibody levels | 9,168,986 | 7763 | European | 2020 | GWAS Catalog |
| GCST90006899 | Epstein-Barr virus EBNA-1  antibody levels | 9,170,056 | 7972 | European | 2020 | GWAS Catalog |
| GCST90006900 | Epstein-Barr virus VCA p18  antibody levels | 9,170,145 | 8518 | European | 2020 | GWAS Catalog |
| GCST90006901 | Epstein-Barr virus ZEBRA  antibody levels | 9,169,747 | 8191 | European | 2020 | GWAS Catalog |
| GCST90006902 | Anti-human herpes virus 6  IgG seropositivity | 9,170,312 | 8735 | European | 2020 | GWAS Catalog |
| GCST90006903 | Anti-human herpes virus 6  IE1A IgG seropositivity | 9,170,312 | 8735 | European | 2020 | GWAS Catalog |
| GCST90006904 | Human herpes virus 6 IE1A  antibody levels | 9,170,460 | 6968 | European | 2020 | GWAS Catalog |
| GCST90006905 | Anti-human herpes virus 6  IE1B IgG seropositivity | 9,170,312 | 8735 | European | 2020 | GWAS Catalog |
| GCST90006906 | Human herpesvirus 6 IE1B antibody levels | 9,171,247 | 7119 | European | 2020 | GWAS Catalog |
| GCST90006907 | Human herpes virus 6 p101k antibody levels | 9,168,031 | 1951 | European | 2020 | GWAS Catalog |
| GCST90006908 | Anti-human herpes virus 7 IgG seropositivity | 9,170,312 | 8735 | European | 2020 | GWAS Catalog |
| GCST90006909 | Human herpes virus 7 U14 antibody levels | 9,171,909 | 8528 | European | 2020 | GWAS Catalog |
| GCST90006910 | Anti-helicobacter pylori IgG seropositivity | 9,170,312 | 8735 | European | 2020 | GWAS Catalog |
| GCST90006911 | Helicobacter pylori CagA antibody levels | 9,165,056 | 985 | European | 2020 | GWAS Catalog |
| GCST90006912 | Helicobacter pylori Catalase antibody levels | 9,167,570 | 1558 | European | 2020 | GWAS Catalog |
| GCST90006913 | Helicobacter pylori GroEL antibody levels | 9,172,299 | 2716 | European | 2020 | GWAS Catalog |
| GCST90006914 | Helicobacter pylori OMP antibody levels | 9,167,440 | 2640 | European | 2020 | GWAS Catalog |
| GCST90006915 | Helicobacter pylori UREA antibody levels | 9,170,248 | 2251 | European | 2020 | GWAS Catalog |
| GCST90006916 | Helicobacter pylori VacA antibody levels | 9,178,635 | 1571 | European | 2020 | GWAS Catalog |
| GCST90006917 | Anti-herpes simplex virus 1 IgG seropositivity | 9,170,312 | 8735 | European | 2020 | GWAS Catalog |
| GCST90006918 | Herpes simplex virus 1 mgG-1 antibody levels | 9,170,062 | 6199 | European | 2020 | GWAS Catalog |
| GCST90006919 | Anti-herpes simplex virus 2 IgG seropositivity | 9,170,312 | 8735 | European | 2020 | GWAS Catalog |
| GCST90006920 | Herpes simplex virus 2 mgG-1 antibody levels | 9,190,612 | 1832 | European | 2020 | GWAS Catalog |
| GCST90006921 | Anti-polyomavirus 2 IgG seropositivity | 9,170,312 | 8735 | European | 2020 | GWAS Catalog |
| GCST90006922 | Polyomavirus 2 JC VP1 antibody levels | 9,171,664 | 5118 | European | 2020 | GWAS Catalog |
| GCST90006923 | Anti-Merkel cell polyomavirus IgG seropositivity | 9,170,312 | 8735 | European | 2020 | GWAS Catalog |
| GCST90006924 | Merkel cell polyomavirus VP1 antibody levels | 9,170,966 | 5915 | European | 2020 | GWAS Catalog |
| GCST90006925 | Anti-Toxoplasma gondii IgG seropositivity | 9,170,312 | 8735 | European | 2020 | GWAS Catalog |
| GCST90006926 | Toxoplasma gondii p22 antibody levels | 9,177,418 | 1308 | European | 2020 | GWAS Catalog |
| GCST90006927 | Toxoplasma gondii sag1 antibody levels | 9,173,429 | 3919 | European | 2020 | GWAS Catalog |
| GCST90006928 | Anti-varicella zoster virus IgG seropositivity | 9,170,312 | 8735 | European | 2020 | GWAS Catalog |
| GCST90006929 | Varicella zoster virus glycoproteins E and I antibody levels | 9,172,177 | 7595 | European | 2020 | GWAS Catalog |

**Table S2.** GWAS data pertaining to five eye diseases

| GWASID | Name | Sample size | ncase | ncontrol | Population | SNPs |
| --- | --- | --- | --- | --- | --- | --- |
| finn-b-H7_IRIDOCHRONIC | CIR | 474964 | 1869 | 473095 | European | 16380407 |
| finn-b-H7_EPISCLERITIS | scleritis | 475056 | 1961/441 | 473095 | European | 16380407 |
| finn-b-DM_RETINOPATHY | DR | 96429 | 14142 | 82287 | European | 16380347 |
| finn-b-WET_AMD | Wet-AMD | 337769 | 6699 | 331070 | European | 16380422 |
| finn-b-H7_GLAUCOMA | Glaucoma | 500348 | 26591 | 473757 | European | 16380466 |

SNPs: single-nucleotide polymorphisms; CIR: chronic iridocyclitis, DR: diabetic retinopathy, Wet-AMD: wet age-related macular degeneration

**Table S3.** Mendelian Randomization Sensitivity Analysis of Antibody-Mediated Immune Responses.

| Outcome | Exposure | PRESSO RSSobs | P_RSSobs_ | Egger intercept | P_Egger_ | Cochrans Q | P_Q_ |
| --- | --- | --- | --- | --- | --- | --- | --- |
| CIR | EBV ZEBRA Ab | 822.6463 | ＜0.001 | 0.061 | 0.094 | 774.344 | 9.236e-112 |
| scleritis | VZV gE & gI Ab | 63.14331 | 0.151 | 0.034 | 0.319 | 59.053 | 0.153 |
| DR | JCV IgG+ | 1680.511 | ＜0.001 | 0.082 | 0.128 | 1509.889 | 1.374e-292 |
| DR | VZV gE & gI Ab | 1610.556 | ＜0.001 | 0.052 | 0.133 | 1462.611 | 4.151e-274 |
| DR | EBV EBNA-1 Ab | 2537.796 | ＜0.001 | -0.015 | 0.574 | 2429.675 | ＜0.001 |
| Wet AMD | JCV IgG+ | 210.776 | ＜0.001 | 0.027 | 0.278 | 196.598 | 2.854e-23 |
| Wet AMD | EBV EBNA-1 Ab | 334.5161 | ＜0.001 | -0.002 | 0.849 | 324.423 | 2.934e-30 |
| Wet AMD | EBV VCA p18 Ab | 246.890 | ＜0.001 | -0.015 | 0.289 | 235.779 | 1.708e-21 |
| Glaucoma | JCV IgG+ | 118.209 | ＜0.001 | 0.006 | 0.503 | 111.995 | 3.195e-09 |
| Glaucoma | EBV EBNA-1 Ab | 301.6406 | ＜0.001 | -7.148e-4 | 0.909 | 291.945 | 4.795e-25 |
| JCV VP1 Ab | DR | 43.50452 | 0.133 | -0.027 | 0.014 | 30.028 | 0.464 |

CIR: chronic iridocyclitis, DR: diabetic retinopathy, Wet-AMD: wet age-related macular degeneration JCV VP1 Ab: Polyomavirus 2 JC VP1 antibody levels, EBV ZEBRA Ab: Epstein-Barr virus ZEBRA antibody levels, VZV gE & gI Ab: Varicella zoster virus glycoproteins E and I antibody levels, JCV IgG+: Anti-polyomavirus 2 IgG seropositivity, EBV EBNA-1 Ab: Epstein-Barr virus EBNA-1 antibody levels, EBV VCA p18 Ab: Epstein-Barr virus VCA p18 antibody levels

**Figure S1.** Funnel plots for the causal effect of antibody-mediated immune responses on five eye diseases.
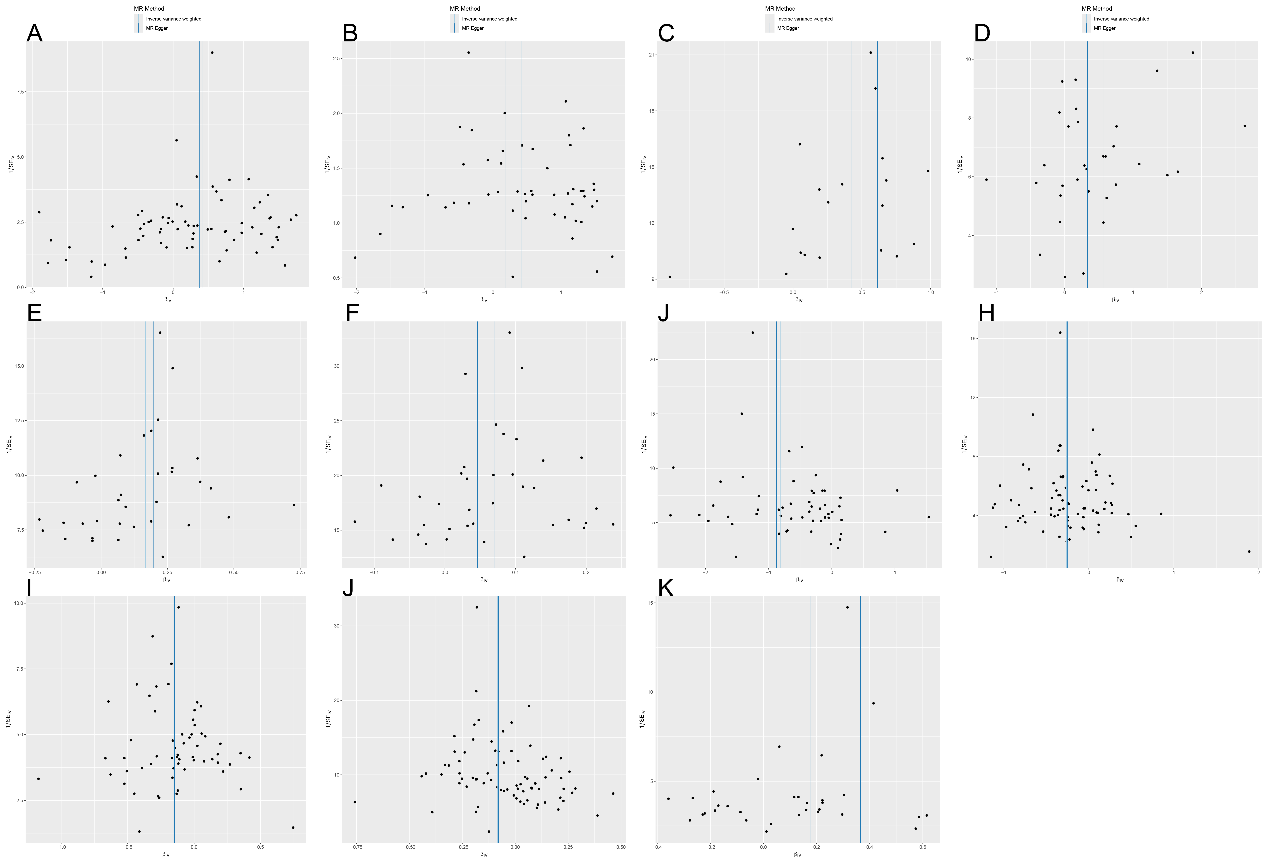


The results of the funnel plot sensitivity analysis were stable and were primarily evaluated using the inverse-variance weighted (IVW) method. (A) Funnel plot depicting the causal association between Epstein-Barr virus (EBV) ZEBRA antibody and chronic iridocyclitis; (B) Funnel plot depicting the causal association between Varicella zoster virus (VZV) glycoprotein E & I antibody levels and scleritis; (C) Funnel plot depicting the causal association between Anti-polyomavirus 2 IgG seropositivity (JCV IgG+) and diabetic retinopathy (DR); (D) Funnel plot depicting the causal association between VZV gE & gI antibody levels and DR; (E) Funnel plot depicting the causal association between JCV IgG+ and wet age-related macular degeneration (AMD); (F) Funnel plot depicting the causal association between JCV IgG+ and glaucoma; (G) Funnel plot depicting the causal association between EBV EBNA-1 antibody levels and DR; (H) Funnel plot depicting the causal association between EBV EBNA-1 antibody levels and wet-AMD; (I) Funnel plot depicting the causal association between EBV VCA18 antibody levels and wet-AMD; and (J) Funnel plot depicting the causal association between EBV EBNA-1 antibody levels and glaucoma; (K) Funnel plot depicting the causal association between DR and JCV VP1 Antibody Levels.

**Figure S2.** Leave-one-out sensitivity analysis for antibody-mediated immune responses on ophthalmic diseases.


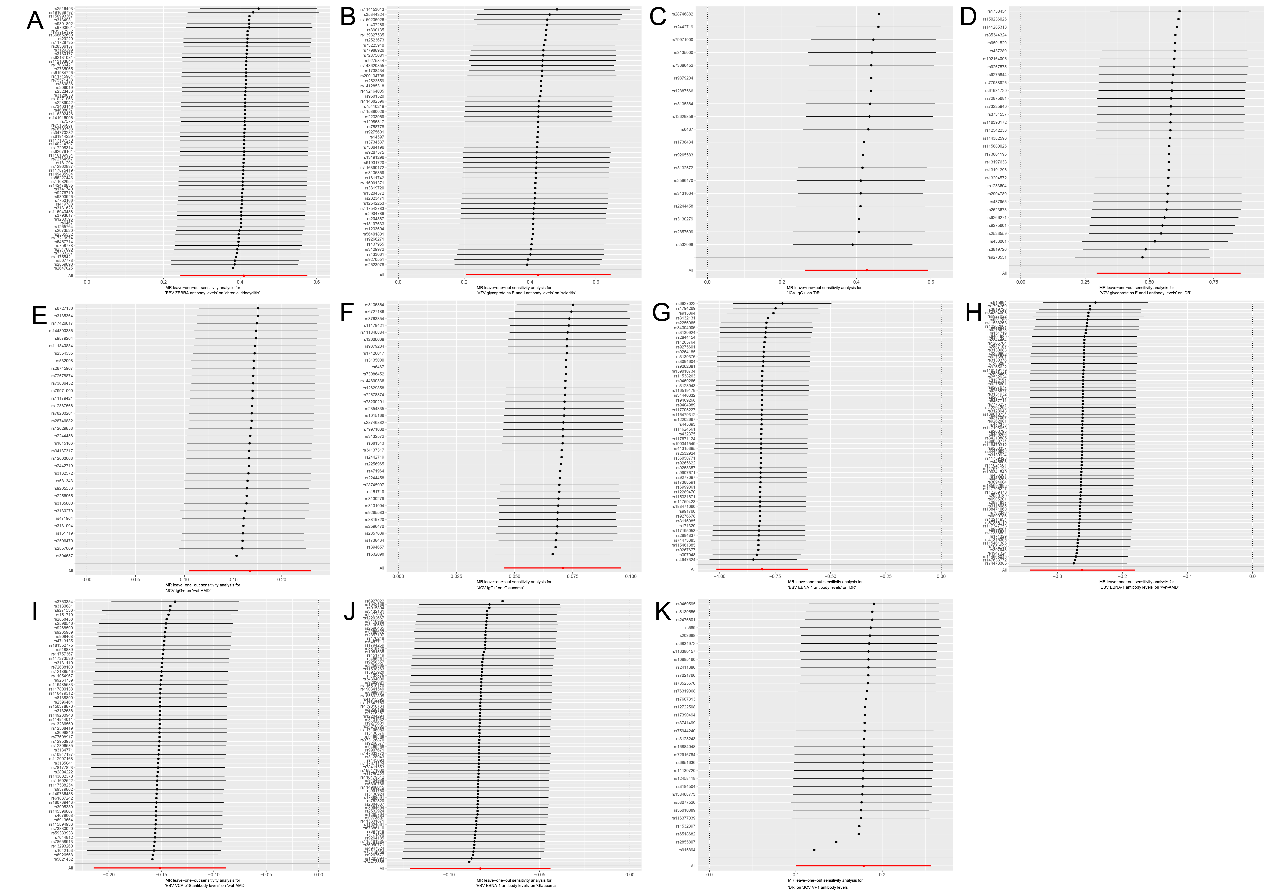


Sensitivity analysis of exposure-related SNPs on outcomes using MR leave-one-out analysis with the IVW method. The x-axis shows the MR analysis results of the remaining SNPs on the outcome after excluding one SNP at a time. (A) Sensitivity analysis for Epstein-Barr virus (EBV) ZEBRA antibody levels on chronic iridocyclitis; (B) Sensitivity analysis for Varicella zoster virus (VZV) glycoprotein E & I antibody levels on scleritis; (C) Sensitivity analysis for Anti-polyomavirus 2 IgG seropositivity (JCV IgG+) on diabetic retinopathy (DR); (D) Sensitivity analysis for VZV gE & gI antibody levels on DR; (E) Sensitivity analysis for JCV IgG+ on wet age-related macular degeneration (AMD); (F) Sensitivity analysis for JCV IgG+ on glaucoma; (G) Sensitivity analysis for EBV EBNA-1 antibody levels on DR; (H) Sensitivity analysis for EBV EBNA-1 antibody levels on wet-AMD; (I) Sensitivity analysis for EBV VCA18 antibody levels on wet-AMD; and (J) Sensitivity analysis for EBV EBNA-1 antibody levels on glaucoma. (K) Sensitivity analysis for DR on JCV VP1 antibody levels.
