# Supplementary figures and images for: Causal effects of antibody-mediated immunity to pathogens on five ophthalmic diseases: a Mendelian randomization study
Source: Exp Biol Med (Maywood). 2026 Jan 30;251:10906. doi: 10.3389/ebm.2026.10906 (PMC12900781; doi:10.3389/ebm.2026.10906)

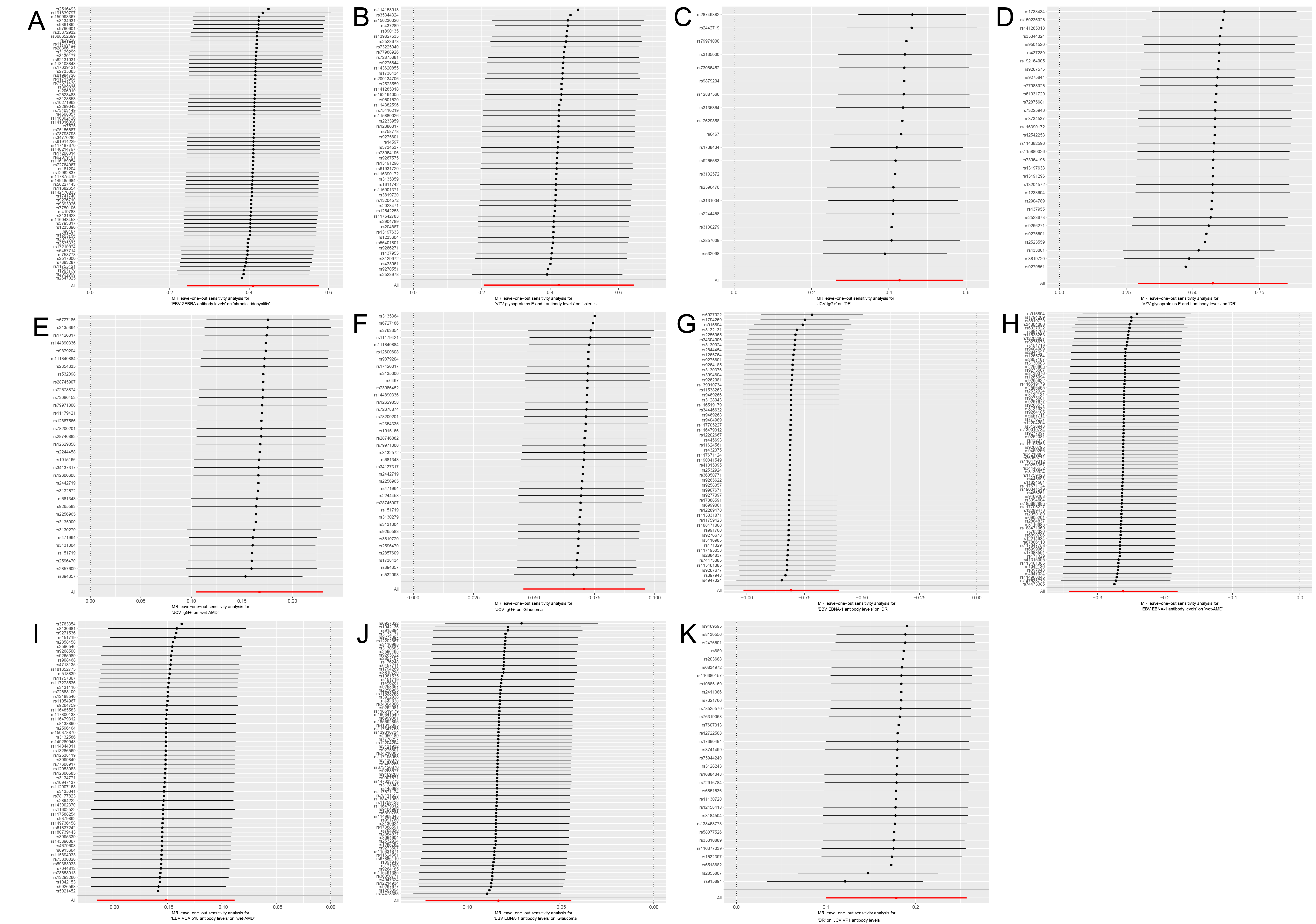

Supplement: Supplementary file 2 [file Image2.tif]

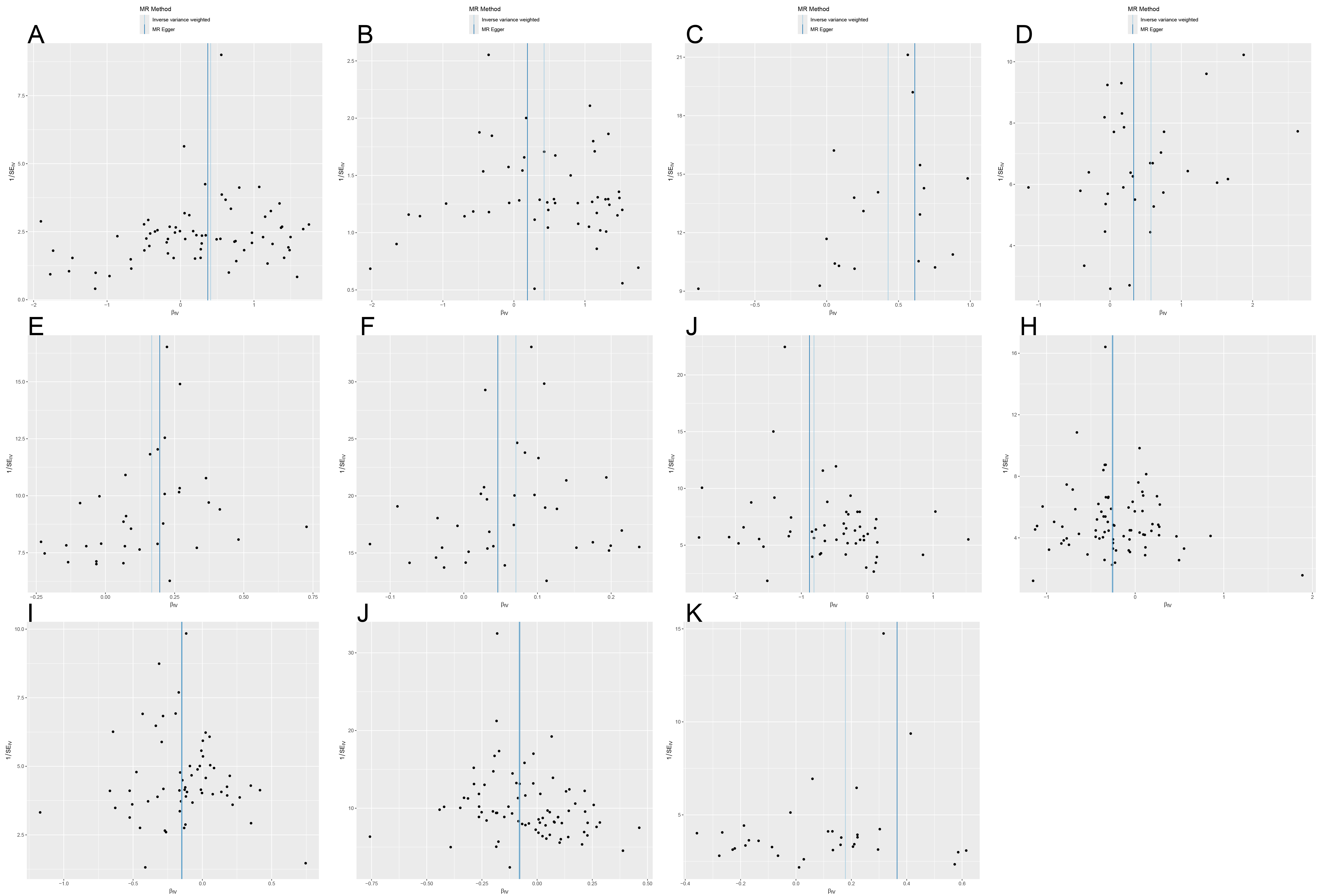

Supplement: Supplementary file 3 [file Image1.tif]
