# Supplementary material for: Causal effects of antibody-mediated immunity to pathogens on five ophthalmic diseases: a Mendelian randomization study
Source: Exp Biol Med (Maywood). 2026 Jan 30;251:10906. doi: 10.3389/ebm.2026.10906 (PMC12900781; doi:10.3389/ebm.2026.10906)
Supplement: Supplementary file 4 [file Table2.docx]

# Expose Mendelian randomisation batch analysis code

# Load necessary R packages

library(TwoSampleMR)

library(ggplot2)

library(foreach)

# ===============================================

# Code Description:

# This code demonstrates a complete Mendelian randomization analysis workflow

# Part 1: Example of clump processing for a single exposure

# Part 2: Batch MR analysis for 46 exposures using pre-clumped CSV files

# ===============================================

# Part 1: Example of clump processing for a single exposure

# Note: This demonstrates how to perform clump processing for one exposure

# In the actual study, this process was repeated 46 times to generate 46 clump files

# These clump files are stored in CSV format in the "clump" folder

# ===============================================

# Set working directory (modify according to actual path)

# setwd("your_working_directory_here")

# Read exposure data (example for a single exposure)

# Note: In actual analysis, each exposure has its corresponding data file

exposure_data <- read.table("SNP_gwas_BK polyomavirus seropositivity.tbl.txt", header = TRUE)

# Correlation filtering (p < 5e-06)

exposure_filtered <- subset(exposure_data, p < 5e-06)

# Save filtered data as CSV format (temporary file for clumping)

write.csv(exposure_filtered, file = "exposure_temp.csv", row.names = FALSE)

# Read exposure data and perform clumping

exp_dat_clumped <- read_exposure_data(

filename = "exposure_temp.csv",

sep = ",",

snp_col = "SNP",

beta_col = "beta",

se_col = "se",

effect_allele_col = "effect_allele",

other_allele_col = "other_allele",

eaf_col = "eaf",

pval_col = "p",

clump = TRUE

)

# Delete temporary file

file.remove("exposure_temp.csv")

# Save clump results as CSV format (example: GCST90006884.csv)

write.csv(exp_dat_clumped, file = "GCST90006884.csv", row.names = FALSE)

cat("Example of single exposure clump processing completed\n")

cat("Generated clump file saved as CSV format: GCST90006884.csv\n")

cat("Note: In actual analysis, we repeated this process 46 times\n")

cat("Generated 46 CSV clump files stored in the clump folder\n")

# ===============================================

# Part 2: Batch MR Analysis

# Description: Batch MR analysis using 46 pre-clumped CSV files

# id.txt file contains the IDs of these 46 exposures

# ===============================================

# Read ID list (containing 46 exposure IDs)

id_df <- read.table("id.txt", header = TRUE, sep = "\t")

exposure_ids <- as.vector(id_df$id)

results_summary <- data.frame()

cat(paste("Starting batch MR analysis for", length(exposure_ids), "exposures...\n"))

# Create results directory

if (!dir.exists("result")) {

dir.create("result", recursive = TRUE)

}

# Create clump directory (if it doesn't exist)

if (!dir.exists("clump")) {

dir.create("clump")

}

# Main loop: MR analysis for each exposure

foreach(current_id = exposure_ids, .errorhandling = "pass") %do% {

# Display current processing progress

current_index <- which(exposure_ids == current_id)

cat(paste0("Processing exposure ", current_index, "/", length(exposure_ids), ": ", current_id, "\n"))

# Construct clump file path (assuming CSV format in clump folder)

clump_file <- paste0("clump/", current_id, ".csv")

# Check if clump file exists

if (!file.exists(clump_file)) {

cat(paste0("Warning: Clump file not found: ", clump_file, ", skipping this exposure\n"))

next

}

# Read clumped exposure data (CSV format)

exposure_data <- tryCatch({

read_exposure_data(

filename = clump_file,

sep = ",",

snp_col = "SNP",

beta_col = "beta.exposure",

se_col = "se.exposure",

effect_allele_col = "effect_allele.exposure",

other_allele_col = "other_allele.exposure",

eaf_col = "eaf.exposure",

pval_col = "pval.exposure"

)

}, error = function(e) {

cat(paste0("Failed to read clump file: ", e$message, "\n"))

return(NULL)

})

if (is.null(exposure_data) || nrow(exposure_data) == 0) {

cat(paste0("Exposure ", current_id, " has no valid data, skipping\n"))

next

}

# Read outcome data

outcome_data <- tryCatch({

read_outcome_data(

snps = exposure_data$SNP,

filename = "finn-b-WET_AMD.gz",

sep = "\t",

snp_col = "rsids",

beta_col = "beta",

se_col = "sebeta",

effect_allele_col = "alt",

other_allele_col = "ref",

eaf_col = "af_alt",

pval_col = "pval"

)

}, error = function(e) {

cat(paste0("Failed to read outcome data: ", e$message, "\n"))

return(NULL)

})

if (is.null(outcome_data) || nrow(outcome_data) == 0) {

cat(paste0("Outcome data for exposure ", current_id, " failed to load, skipping\n"))

next

}

# Data harmonization

harmonized_data <- harmonise_data(

exposure_dat = exposure_data,

outcome_dat = outcome_data,

action = 2

)

if (nrow(harmonized_data) == 0) {

cat(paste0("No SNPs after harmonization for exposure ", current_id, ", skipping\n"))

next

}

# Calculate R² and F-statistics

harmonized_data$R2 <- (2 * (harmonized_data$beta.exposure^2) * harmonized_data$eaf.exposure * (1 - harmonized_data$eaf.exposure)) /

(2 * (harmonized_data$beta.exposure^2) * harmonized_data$eaf.exposure * (1 - harmonized_data$eaf.exposure) +

2 * harmonized_data$samplesize.exposure * harmonized_data$eaf.exposure * (1 - harmonized_data$eaf.exposure) * harmonized_data$se.exposure^2)

harmonized_data$f <- harmonized_data$R2 * (harmonized_data$samplesize.exposure - 2) / (1 - harmonized_data$R2)

harmonized_data$meanf <- mean(harmonized_data$f)

# Filter weak instrumental variables (F-statistic > 10)

harmonized_data <- harmonized_data[harmonized_data$f > 10, ]

if (nrow(harmonized_data) == 0) {

cat(paste0("Exposure ", current_id, " has no SNPs with F-statistic > 10, skipping\n"))

next

}

# Initialize processed data

data_for_mr <- harmonized_data

# MR-PRESSO analysis to detect outlier SNPs

mr_presso_result <- NULL

tryCatch({

mr_presso_result <- run_mr_presso(harmonized_data, NbDistribution = 1000)

# Extract outlier indices

outlier_index <- mr_presso_result[[1]]$`MR-PRESSO results`$`Distortion Test`$`Outliers Indices`

# If outlier SNPs exist, remove them

if(!is.null(outlier_index) && length(outlier_index) > 0) {

outlier_snps <- harmonized_data[harmonized_data$new_id %in% outlier_index, "SNP"]

cat(paste("Detected outlier SNPs:", paste(outlier_snps, collapse = ", "), "\n"))

# Remove outlier SNPs

data_for_mr <- harmonized_data[!harmonized_data$new_id %in% outlier_index, ]

cat(paste("Removed", length(outlier_index), "outlier SNPs\n"))

} else {

cat("No outlier SNPs detected\n")

}

}, error = function(e) {

# If MR-PRESSO fails, continue with original data

cat(paste("MR-PRESSO error:", e$message, "\n"))

data_for_mr <- harmonized_data

})

# Perform MR analysis with processed data

mr_results <- mr(data_for_mr)

odds_ratios <- generate_odds_ratios(mr_results)

# Get IVW method results (typically row 3)

ivw_index <- which(mr_results$method == "Inverse variance weighted")

if (length(ivw_index) == 0) {

cat(paste0("Exposure ", current_id, " has no IVW method results\n"))

next

}

# If MR results are significant (IVW method, p < 0.05), save detailed results

if (mr_results$pval[ivw_index] < 0.05) {

# Record results to summary table

results_summary <- rbind(results_summary,

data.frame(id = current_id,

pvalue = odds_ratios$pval[ivw_index]))

# Create individual result directory

result_dir <- paste0("result/", current_id)

if (!dir.exists(result_dir)) {

dir.create(result_dir, recursive = TRUE)

}

# Save MR-PRESSO results

if (!is.null(mr_presso_result)) {

capture.output(mr_presso_result, file = paste0(result_dir, "/mr_presso_results.txt"))

}

# Save harmonized data

write.table(data_for_mr,

file = paste0(result_dir, "/harmonise.txt"),

row.names = FALSE, sep = "\t", quote = FALSE)

# Save OR results

write.table(odds_ratios[, 5:ncol(odds_ratios)],

file = paste0(result_dir, "/OR.txt"),

row.names = FALSE, sep = "\t", quote = FALSE)

# Pleiotropy test

pleiotropy <- mr_pleiotropy_test(data_for_mr)

write.table(pleiotropy,

file = paste0(result_dir, "/pleiotropy.txt"),

sep = "\t", quote = FALSE, row.names = FALSE)

# Heterogeneity test

heterogeneity <- mr_heterogeneity(data_for_mr)

write.table(heterogeneity,

file = paste0(result_dir, "/heterogeneity.txt"),

sep = "\t", quote = FALSE, row.names = FALSE)

# Scatter plot

scatter_plot <- mr_scatter_plot(mr_results, data_for_mr)

ggsave(scatter_plot[[1]],

file = paste0(result_dir, "/scatter.pdf"),

width = 8, height = 8)

# Single SNP analysis

single_snp_results <- mr_singlesnp(data_for_mr)

single_snp_or <- generate_odds_ratios(single_snp_results)

write.table(single_snp_or,

file = paste0(result_dir, "/singlesnpOR.txt"),

row.names = FALSE, sep = "\t", quote = FALSE)

# Forest plot

forest_plot <- mr_forest_plot(single_snp_results)

ggsave(forest_plot[[1]],

file = paste0(result_dir, "/forest.pdf"),

width = 8, height = 8)

# Leave-one-out sensitivity analysis

leaveoneout_results <- mr_leaveoneout(data_for_mr)

sensitivity_plot <- mr_leaveoneout_plot(leaveoneout_results)

ggsave(sensitivity_plot[[1]],

file = paste0(result_dir, "/sensitivity_analysis.pdf"),

width = 8, height = 8)

# Funnel plot

funnel_plot <- mr_funnel_plot(single_snp_results)

ggsave(funnel_plot[[1]],

file = paste0(result_dir, "/funnelplot.pdf"),

width = 8, height = 8)

cat(paste0("Exposure ", current_id, " MR results significant, detailed results saved\n"))

} else {

cat(paste0("Exposure ", current_id, " MR results not significant (p=",

round(mr_results$pval[ivw_index], 4), ")\n"))

}

}

# Save overall results

write.table(results_summary,

file = "overall_results.txt",

sep = "\t",

quote = FALSE,

row.names = FALSE)

cat(paste0("Batch MR analysis completed!\n"))

cat(paste0("Number of significant results: ", nrow(results_summary), "\n"))

cat(paste0("Overall results saved to: overall_results.txt\n"))
